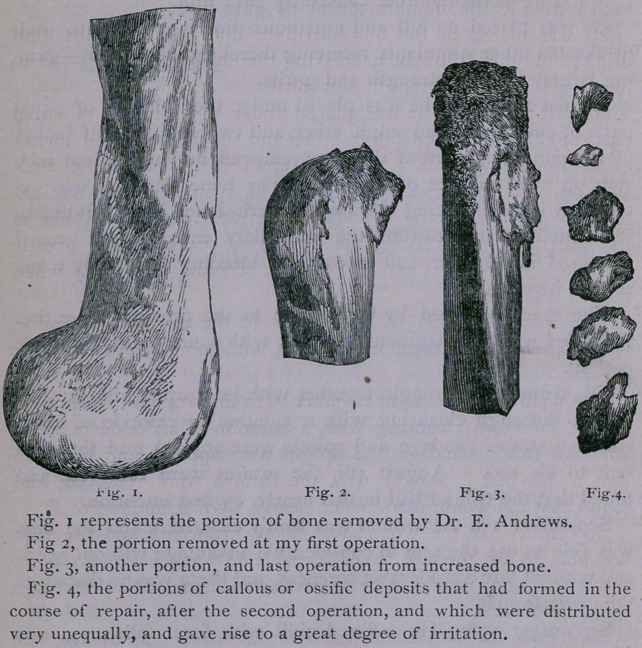# Excisions of Head of Humerus for Gun Shot Wounds

**Published:** 1871-06

**Authors:** Charles M. Clark

**Affiliations:** Late Surgeon 39th Ill. Vol. Infantry, and Chief Operating Surgeon 24th Army Corps


Article II.—*Excisions of Head of Humerus for Gun Shot Wounds.* By Charles M. Clark, M.D., late Surgeon 39th Ill. Vol. Infantry, and Chief Operating Surgeon 24th Army Corps.

CASE I.

George Parce, private, company B, 44th Regiment Ill. Vols., wounded at the battle of Chicamauga, September 20th, 1863, by conoidal bullet, which entered anterior surface of arm, four inches below the head of bone, passing almost transversely through the soft tissues, impinging slightly on the shaft of humerus during its passage.

No treatment, save simple cold water dressing, was applied or considered necessary at the time.

He entered the "Chicago Soldiers' Home" in the month of January, 1865, with caries and necrosis of the upper third of the humerus, involving the head of the bone. There was free discharge of pus from four distinct sinuses on the dorsal surface of the arm, and he complained of constant pain.

An operation for excision of the head of bone was performed April 7th, 1865, by Dr. E. Andrews of this city, when the head of the humerus and a portion of its shaft was removed, the whole measuring five and one-fourth inches in extent.

The wound of the operation did not heal readily, chiefly on account of the great impoverishment of the general system; erysipelas occurring, which run its course, and was attended with considerable sloughing.

The case came under my notice June 16th, 1865, at which time I took surgical charge of the Home. The arm was then swollen and painful, with discharge of a sanious pus, and a large extent of denuded bone was detected with the probe. The patient was anxious for another operation, saying that he could not survive such torture as the member constantly gave him.

He was placed on full and nutritious diet, together with malt drinks and other stimulants, receiving thereby great benefit—gaining largely in flesh, strength and spirits.

August 2nd, 1867, he was placed under the influence of equal parts of chloroform and sulph. ether, and two and one-half inches of the remaining shaft of the bone removed by longitudinal incision on outer surface of the arm. The bone removed was extensively diseased, being denuded of periosteum, and shell-like in its structure and calibre, the medullary canal being greatly increased in diameter, and its contents bleeding very freely when section was made.

The space occupied by bone prior to the previous operation was filled with cartilaginous material, with some points of ossific deposit.

The wound was brought together with interrupted silk sutures, after a thorough cleansing with a solution of chloride of zinc; adhesive straps, bandage and splints were applied, and the man sent to his bed. August 4th, the sutures were removed, and found that the wound had healed nearly, by first intention.

September 1st, the wound was firmly cicatrized, and the man was able to use the arm to considerable advantage without pain, and without any considerable soreness, and there was no discharge whatever from it.

September 15th—He suffered chill with fever following, and from this date the wound commenced to inflame, and soon discharged large quantities of pus from two different sinuses. The probe detected a large surface of dead bone, and, with his consent, another operation was decided upon, and which was performed October 3d, 1867, when three and one-half inches more of the bone were removed, making eleven and one-fourth inches in all.

The portion removed presented, almost entirely, the same characteristics as the portion of bone previously removed.

During the operation, whatever portion of the periosteum was in contact with the bone was dissected off, for the purpose of testing its capacity for reproduction.

The wound healed rapidly, and ceased discharging, with no other dressing than a solution of chloride of zinc, frequently applied to the parts with a sponge.

Fig. 1. Fig. 2. Fig. 3. Fig. 4.

Fig. 1 represents the portion of bone removed by Dr. E. Andrews.

Fig 2, the portion removed at my first operation.

Fig. 3, another portion, and last operation from increased bone.

Fig. 4, the portions of callous or ossific deposits that had formed in the course of repair, after the second operation, and which were distributed very unequally, and gave rise to a great degree of irritation.

In March, 1868, he could use the arm to great advantage—feed himself with it, and carry it up sufficiently high to take off his hat.

He left the "Home" in the latter part of March, 1868, feeling well, and had gained some twenty pounds in flesh.

I have seen this man twice within the past two years (1869–70), and he is vigorous and healthy. The arm has not troubled him since the last operation, and is filling up with a cartilaginous substance that grows firmer every day.

The last time I saw him he was complaining of some trouble connected with his heart, and was confident of some lesion there from the repeated shocks to his nervous system, occasioned by the frequent use of chloroform and the knife.

The depression under the acromion was disappearing, and he could use the arm to a considerable extent without aid from the other arm. There was a condition of anchylosis at the shoulder, and when the arm was raised, a false-joint was manifest at the middle third.

This case presents several features that are full of interest and instruction to the surgeon.

1st. The amount of bone removed (eleven and one-fourth inches), and the subsequent usefulness of the arm.

2d. The condition of the medullary canal and its contents (as noticed at the second and third operations), with the degree of bone absorption that had taken place, leaving the remaining shaft of the bone a mere shell.

3d. The disposition of bone tissue to reproduce itself, as witnessed in the portions removed, showing a tendency to heal and cover in the medullary. In each section of bone removed, after the first operation, there is noticeable a growth in the shaft, from the point of section to the extent of from a quarter to half an inch in extent, and, aside from this, there was a disposition in some portions of the shaft to throw out callus to heal the parts that had been encroached upon by disease.

If it is the function of the periosteum to nourish and reproduce bone, why is there not some uniformity in the process?—not one deposit here and another there, which instead of helping along repair, only creates more trouble from the irritation produced.

In all amputations there is noticed a new development of bone, extending, in some cases that I have seen, to the extent of three-quarters of an inch, and then terminating in a bulbous expansion, only limited by the length of the stump.

### CASE II.

Joseph La Rock, private, company C, 9th Vermont Vol., aged 40 years.

This man received an accidental gunshot wound May 12th, 1865, at Richmond, Va., the ball (conoidal) entering right pectoral muscle at its outer third, passing through the capsular ligament of the shoulder joint, and extensively fracturing the head of the bone, the ball making its exit at middle of upper third of arm (the arm being at right angle with body).

May 13th—He was placed under the influence of chloroform at the 24th Army Corps hospital, and the following operation performed. An incision was commenced at the acromion process, and carried down on the dorsum of arm to the extent of five inches, through to the bone. An assistant then kept the edges of wound separated, while I removed the fragments and spicula of bone, being careful to denude them of periosteum.

The next step in the operation was to remove the head of the bone, which was easily accomplished with a few clips of the knife; then, after cutting away the ragged portions of the capsule, I paid attention to the lower portion of the humerus, and section made at a point where the shaft was complete—some two and one-half inches from the head of the bone.

The wound was brought together with sutures and adhesive straps, and compress and cold water dressing applied.

The man was put to bed, and remained under my care until perfectly recovered—some six weeks time—and then was mustered out of service by reason of disability.

The wound in this case healed rapidly and by the first intention in two thirds of its extent; the lower third was more slow in healing, and discharged continually a laudable pus in moderate degree, and healed by granulation.

At the time of his discharge he could raise the arm to a right-angle with his body, and the forearm could be used in lifting the cap from his head, and in feeding himself.

There was no atrophy or wasting away of the tissues of the arm; the member, after his discharge, being as plump and well developed as before the operation, for the reason, perhaps, that he had been growing more stout while an inmate of the hospital.

At the time I last examined the arm I found it to be firm, with a cartilaginous deposit, which was undergoing a change, as bony deposits could be distinctly felt; and if the man has taken sufficient care of himself since his discharge, he has a useful ar m.

(To be continued.)

## Figures and Tables

**Fig. 1. Fig. 2. Fig. 3. Fig. 4. f1:**